# Dynamics of human categorization in a collaborative tagging system: How social processes of semantic stabilization shape individual sensemaking

**DOI:** 10.1016/j.chb.2015.04.053

**Published:** 2015-10

**Authors:** Tobias Ley, Paul Seitlinger

**Affiliations:** a Tallinn University, Tallinn, Estonia; b Graz University of Technology, Graz, Austria

**Keywords:** Categorization, Sensemaking, Collaborative tagging, Distributed cognition, Social web

## Abstract

We study how categories form and develop over time in a sensemaking task by groups of students employing a collaborative tagging system. In line with distributed cognition theories, we look at both the tags students use and their strength of representation in memory. We hypothesize that categories get more differentiated over time as students learn, and that semantic stabilization on the group level (i.e. the convergence in the use of tags) mediates this relationship. Results of a field experiment that tested the impact of topic study duration on the specificity of tags confirms these hypotheses, although it was not study duration that produced this effect, but rather the effectiveness of the collaborative taxonomy the groups built. In the groups with higher levels of semantic stabilization, we found use of more specific tags and better representation in memory. We discuss these findings with regard to the important role of the information value of tags that would drive both the convergence on the group level as well as a shift to more specific levels of categorization. We also discuss the implication for cognitive science research by highlighting the importance of collaboratively built artefacts in the process of how knowledge is acquired, and implications for educational applications of collaborative tagging environments.

## Highlights

• We study how categories people develop in collaborative tagging change over time.
• Their internal cognitive categories and the tags they use are coordinated.
• Especially groups converging in the use of terms develop differentiated categories.
• Social processes around shared artefacts have a mediating effect on learning.

## 1 Introduction

Because of the ubiquity of social web technologies, there has been a recent growing interest in how people make sense of large quantities of information when they browse the web (Pirolli & Russell, 2011). In this paper, we focus on the sensemaking process which results from people using a collaborative tagging system (Golder & Huberman, 2006; Marlow, Naaman, Boyd, & Davis, 2006). In systems like *Delicious* (websites), *Flickr* (photos) or *Soundcloud* (music), people describe different types of resources they discover on the web by assigning freely chosen keywords (called *tags*) to store them for later use. Fu (2008) describes this process as an iterative exploratory search-and comprehend cycle which leads to a close interaction between internal and external representations of concepts, tags, and resources as a user searches the web. The tags that a user applies are a result of his or her mental categorization processes. Over time and as more resources are tagged, a user’s understanding of a particular topic increases, and his internal categories change and become more refined.

Because the resources and tags collected by one user can be seen by others, it is usually assumed that individuals are influenced by tags as social cues. In the social web, there is considerable influence of collective information on individual behavior (Li & Sakamoto, 2014). In collaborative tagging, tags function as primes in activating prior knowledge (Cress, Held, & Kimmerle, 2013; Fu, 2008), users imitate each other’s tag assignments to a certain degree (Fu, Kannampallil, Kang, & He, 2010; Seitlinger & Ley, 2012; Seitlinger, Ley, & Albert, 2015; Sen et al., 2006), and learning processes can take place when a user browses the tag collection of another user, thereby discovering resources and tags that influence that user in his/her future tag assignments (Held, Kimmerle, & Cress, 2012; Nelson et al., 2009). As a consequence, collaborative tagging allows for studying how individual sensemaking is shaped by social processes, namely by artefact-mediated collaboration.

Gaining an understanding of the development of categories and the corresponding tag assignments is important for improving information access in the social web. Research on improving information access with tags has been seeking different routes. For example some researchers have suggested enhancing semantic search technologies with social tags (e.g. Gayo, de Pablos & Lovelle, 2010; Specia & Motta, 2007). Another direction has been to employ recommender services that are based on tags, or that suggest suitable tags (Bollen & Halpin, 2009; Jäschke, Marinho, Hotho, Schmidt-Thieme, & Stumme, 2007). Finally, several tools have been suggested that attempt to enhance the collaboration processes around tags and their corresponding categories (e.g. Braun, Schmidt, Walter, Nagypal, & Zacharias, 2007; Tolosa, Gayo, Prieto, Núñez, De Pablos, 2010; Yew, Gibson, & Teasley, 2006).

However, to fully exploit this potential for semantic technologies or recommenders, it is important to understand the process by which shared terminological patterns can emerge without an explicit coordination (Gayo et al., 2010). This is because retrieval of information may actually become more difficult both for experts and for novices when resources are described on varying levels of specificity. While for the former, the information value of a basic level category is too low, for the latter the specific categories are not sufficiently well represented in memory, and, hence, their labels difficult to comprehend (Rogers & Patterson, 2007).

The particular contribution of our paper is twofold. First, and rather than studying the information access, we examine the underlying mechanisms of how people develop and extend their categorization in collaborative tagging system. Our assumption is that phenomena of categorization can only be studied by looking at how internal categories and tags people use are coupled. Therefore, we looked both at internal categories students learned, as well as the tags they used.

Secondly, we study how the development of categories is mediated by an important social process in collaborative tagging, namely the development of shared language that results from semantic stabilization (e.g., Baronchelli, Felici, Loreto, Caglioti; Crokidakis & Brigatti, 2015; Steels, 2006). Here, we hypothesize that individual sensemaking will be more successful if it is built on a shared use of tags on the group level. While in previous studies, the impact of tags as social cues on individual learning and browsing behavior was examined under lab conditions (e.g. Held et al., 2012), none of these previous studies has actually looked at how semantic stabilization influences individual learning. This is because semantic stabilization is a social process that develops over time and therefore needs to be studied in a setting that allows users to interact with each other over a more extended period of time. Others have examined the development of categories when using a tagging system (Fu & Dong, 2012), but have not focused on semantic stabilization as a mediating factor.

We have therefore conducted a field experiment in which students used a collaborative tagging system in the context of a university course over a more extensive period of time. By giving some groups more time for their task, we realized a condition in which semantic stabilization should be more likely as compared to those groups that spent less time working on the task. As a measure of individual learning, we then looked at the categories and their increasing specificity that students developed as a result, in terms of both, the tags students used and their internal memory representation.

## 2 Dynamics of human categorization in collaborative tagging

When students interact with the Collaborative Tagging system by searching for web resources, assigning tags and sharing these with other students, we assume a dynamic coupling between the students and their shared artifacts forming a cognitive ecosystem (Hutchins, 2010). The artifacts (e.g. tags) influence students’ categorization and – depending on the artifacts’ emerging shape and structure – support or exacerbate the collaborative process of exploring and deepening the understanding of a given topic. Similar to individual learning progress, where internal categories do not emerge out of nowhere but are differentiated representations of existing ones (e.g., Rogers & Patterson, 2007), artifacts emerge over time from a collective artifact-mediated activity (Hutchins & Hazlehurst, 1995). Here, we use the term artifact to refer to some type of inscription (Latour & Woolgar, 1986) that is a malleable means of representation of things (e.g., Web resources) that can be changed and improved continuously by members of a community (e.g., students of a university course) (see also Schreiber, 2006). The continuous development of artifacts, such as tags assigned to a Web resource, reflects the ongoing development of underlying processes of understanding of things within the community and thereby provides an empirical unit of analysis. The relationship between artifacts and things to which they refer, becomes a matter of a dynamic, social practice (e.g., Roth & McGinn, 1998; see also Schreiber, 2006), leading to a shared understanding of this relationship.

In case of tagging, one simple mechanism behind the emergence of a shared artifact-thing-relationship is semantic priming: existing tags prompt a particular user to activate related memory content resulting in converging categorization and verbalization processes among the users (e.g., Fu & Dong, 2012; Fu et al., 2010; Seitlinger et al., 2015). Over time, these mutual influences create positive feedback loops (Hutchins & Johnson, 2009) that result in semiotic dynamics (e.g., Steels, 2006) giving rise to semantic stabilization (Wagner, Singer, Strohmaier, & Huberman, 2014), i.e., a consensual use of tags for a resource. In terms of distributed cognition, the mechanism of a positive feedback loop underlying the spreading adoption of tag-resource pairs can be described as follows: “Once a behavior enters the repertoire of one agent, …, it is likely to enter the repertoires of others, which makes it even more likely to enter the repertoires of still others, and so on.” (Hutchins & Johnson, 2009, p. 526).

The assumed semiotic feedback loops leading to semantic stabilization imply that external artifacts and peoples’ interpretations co-shape each other, as also proposed by approaches towards cognition distributed across extended systems of human and non-human actants (e.g., Fu, 2008; Hutchins, 2010; Hutchins & Hazlehurst, 1995; Latour, 1996): Artifacts introduced by preceding individuals augment the experience of subsequent individuals; the augmented experience may influence its interpretation and hence, the creation of new artifacts.

To summarize, our main assumption is that different levels of semantic stabilization that form in a group observable in the use of tags will mediate individual understanding in a sensemaking task. In the following two sections, we will first discuss how semantic stabilization has been studied in collaborative tagging and define a way to measure it. Secondly, we will introduce the basic level shift in categorization as a way to define a deepening of understanding in individual sensemaking. We will then summarize our hypotheses in more detail and describe a field experiment that was designed to test the influence of semantic stabilization on individual sensemaking.

### 2.1 Semantic stabilization in collaborative tagging: the emergence of a shared language

Although all users of a collaborative tagging system are free to use whichever keyword they want to describe the resources they collect, research in the tradition of semiotic dynamics (e.g., Steels, 2006) has shown that the development of a consistent tag vocabulary can usually be observed (Golder & Huberman, 2006). In the present study, we measure an emerging coherence in tag assignments by observing the time evolution of the number of unique tags *N_u_* (see also Baronchelli, Felici, Loreto, Caglioti, & Steels, 2006). Drawing *N_u_* against consecutive tag assignments results in a curve that visualizes a specific aspect of semiotic dynamics, namely the convergence time (e.g., Steels, 2006), which is the time needed to reach a stable level of coherence within the shared set of tags.

Usually, the distribution follows an exponential function (e.g., Golder & Huberman, 2006). Since simple stochastic models, such as Polya’s urn model, provide a good account of the emerging stabilization in tagging systems (e.g., Golder & Huberman, 2006; Wagner et al., 2014), we assume that the time-dependent semantic stabilization follows an exponential decay function (e.g., Bousfield & Sedgewick, 1944) given by

(1) $$N_{u}(\text{t})=H*(1-e^{-\mathrm{bt}})$$

The function provided by Eq. (1) exhibits two features: the asymptote *H*, which is the estimated stabilization given unlimited time, and the slope *b* measuring the rate of approaching the asymptote. To address the first question of operationalization we make use of both parameters to characterize semantic stabilization in terms of the general level of stabilization, *H*, and in terms of the speed of convergence, *b*.

### 2.2 Sensemaking and the differentiation of categories

As discussed above, sensemaking when using the web is a process in which internal categories are refined in an iterative manner as understanding in a topic increases. This assumption is based on robust findings from research on human categorization taking place at several levels of abstraction (e.g., Close & Pothos, 2012). Typically, three levels of abstraction of how humans describe objects are differentiated, i.e. a *superordinate level* (e.g., plant), a *basic level* (e.g., tree) and a *subordinate level* (e.g., fir). The seminal works of Rosch, Mervis, Gray, Johnson and Boyes-Braem (1976; see also Rogers & Patterson, 2007) has shown that people prefer basic-level categories over super- and subordinate categories to categorize and name objects of the environment. In line with this robust basic-level advantage is the observation that the first tag a user assigns to a Web resource usually represents a basic-level category and that consensus among users usually emerges around such basic-level tags (Golder & Huberman, 2006). This observation is in line with our assumption of a dynamic coupling of internal categories with the created artefacts (tags), as internal categories seem to be coordinated with manifestations of tags in collaborative tagging systems.

As the expertise of a person within a particular topic increases, subordinate categories become more differentiated and more easily available in categorization and naming tasks (e.g., Tanaka & Taylor, 1991). Thus, this so-called basic-level shift is indicative for a person’s expertise and the frequency of applying as well as the strength of representations of subordinate categories should provide a measure to distinguish between people who differ in the understanding of a given topic. For the setting of the present study, we assume that students gaining a deeper understanding of a topic should exhibit a stronger basic-level shift than students with a more shallow understanding. A stronger basic-level shift should become manifest in a frequent use and in a strong internal representations of tags for subordinate categories.

In our study, each group of students was instructed to collaboratively generate a hierarchy of tags by means of a taxonomy editor available in the shared Web environment. For measuring the basic level shift, we draw on these taxonomies. Specifically, we regard tags of the first level as basic-level tags and tags of the levels below as subordinate tags (see the Method section for a validation check of the assumption that different taxonomy levels correspond to different levels of abstraction).

### 2.3 Hypotheses

As described above, we assume that learning in a shared environment is mediated by shared artifacts, in particular by the extent to which these artifacts support a collaborative exploration and understanding of a topic. For instance, if students succeed in using similar tags for certain aspects of a topic, then subordinate levels of the tag taxonomy should provide helpful tags to categorize newly found Web resources. As a consequence, these subordinate tags should be frequently used and represented in form of strong, internal memory representations. Therefore, we hypothesize an interdependence between attributes of semiotic dynamics on the group level, i.e., *H* and *b*, and students’ basic level shift. *Students of groups performing a higher level and faster rate of semantic stabilization within their shared tag vocabulary should – on average – exhibit a more frequent use and a stronger internal representation of subordinate tags than students of groups with a lower level and slower rate of semantic stabilization*.

As our first hypothesis, we therefore assume the following:

H1 Users of a collaborative tagging system will develop a more common understanding of the concepts named by the tags when they collaboratively tag for a longer as compared to a shorter duration of time.

This means that semantic stabilization should be more pronounced in groups that tag resources for a topic a longer as compared to a shorter amount of time. We measured this convergence time by comparing the number of different tags over time between groups with a short vs. a long engagement with a topic.

As the second hypothesis we then assume

H2 Students in groups with a higher level and faster rate of semantic stabilization within their shared tag vocabulary should – on average – exhibit a more frequent use (hypothesis H2.1) and a stronger internal representation (hypothesis H2.2) of subordinate tags than students of groups with a lower level and slower rate of semantic stabilization.

## 3 Method

To test these hypotheses, it was necessary to observe a group of users that naturally use a collaborative tagging system for a sensemaking task over some period of time. We also needed to collect additional data from those users, such as data about the representation of tags in their memory. Therefore, a university course setting provided an adequate setting for conducting our study, as we were able to randomly assign students to groups, give them rather realistic tasks and still control their engagement to a certain degree.

For this reason, we chose to conduct a field experiment in which we asked students to collaboratively collect bookmarks related to their course subject and describe them with tags within the shared bookmarking system SOBOLEO. Additionally, by means of the SOBOLEO taxonomy editor, the students had to specify super- and sub-relations between their tags resulting in a collaborative hierarchy of tags with different abstraction levels. That way, we allowed students’ categorization behavior to emerge naturally. At the same time, we introduced an experimental design controlling for unwanted effects, such as the fact that students had to learn to use SOBOLEO at the same time that they were learning about a topic. How exactly the experimental design controlled for these effects will be described in more detail in Section 3.3.

### 3.1 Participants

The study took place in the context of a university course on cognitive models in technology enhanced learning at the University of Graz that was conducted in the autumn term 2009/2010. Participants (*N = *25, 12 female) were psychology students participating for course credit. Alternatively, they could choose writing an essay for the same course credit, but each of them preferred participating in the study. The average age was 23.3 years (SD = 1.2) ranging from 21 to 25 years.

### 3.2 Materials

#### 3.2.1 SOBOLEO: A collaborative tool for bookmarking, tagging and taxonomy creation

**Fig. 1:**
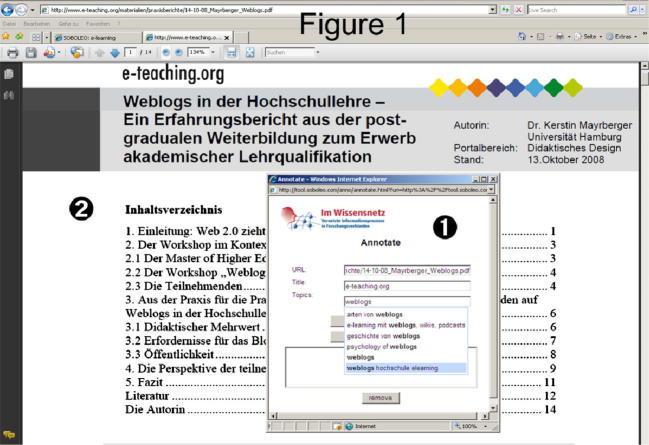
The annotation widget of SOBOLEO.

To collaboratively work on course-related topics the students made use of the *social bookmarking system SOBOLEO* (http://mature-ip.eu/tool/soboleo; Braun et al., 2007). Here, users annotate bookmarks individually with tags, and then use the tags to build a shared vocabulary and a taxonomic structure. Fig. 1 shows a screenshot of the annotation widget. When discovering a website with their web browser (2), a person can open an annotation widget and type a number of tags to describe the website (1), and then store the tagged bookmark on a server. All bookmarks and tags created by a person are visible to all other users as well. As the user types in a tag, the annotation widget suggests all tags that have already been used by anyone within the group by displaying in a list all tags that start with the sequence of letters the user has started typing.

**Fig. 2:**
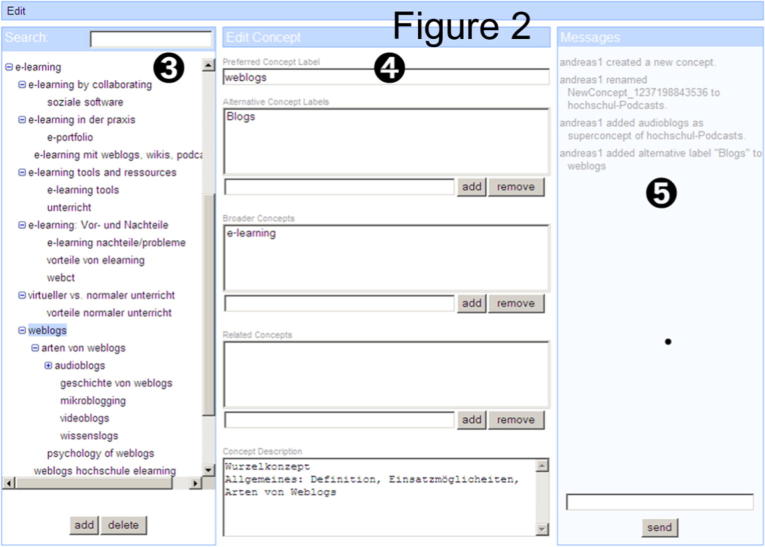
The SOBOLEO taxonomy editor.

Fig. 2 shows the SOBOLEO taxonomy editor that displays the shared tags. The taxonomy can be built collaboratively by all students in the group. Each person can drag and drop tags (which are initially sorted under *“prototypical concepts”*) to the taxonomy tree in the editor (3), and enter textual labels (4). These changes are reflected in the system for all users and are therefore visible to all in the same way. A chat (5) is available for discussing decisions on moving a tag to one branch of the tree rather than another, or about labeling. In the current study, the chat could not be used for technical reasons. Instead, a discussion forum was provided through the WebCT course environment. Here the groups were invited to discuss the building of the shared taxonomy.

The use of the taxonomy editor in SOBOLEO allowed us to study the specificity of tags by drawing the tag samples from different levels down the branches of the taxonomies created by the students (see Section 3.4 on tag samples below). The process of collaboratively creating a taxonomy is a specificity of the SOBOLEO system not available in most current tagging systems. This functionality should enhance the common understanding of the tags among the users and improve the overall consistency of the tag collection. While this is a variation of the usual practice of collaborative tagging, we still decided to use the taxonomy editor in our study for the following reasons. First, this allowed us to derive a measure of tag specificity independent of the use of the tags in the tag assignments. Algorithms that derive the implicit relations between tags (e.g. from their co-occurrence) are not independent from tag frequency measures, and would therefore be problematic for our purposes. Secondly, as the study duration was relatively short (as compared to usual usage durations in collaborative tagging systems) we were intending to enhance intentional collaboration by this mechanism.

#### 3.2.2 Free association test: Eliciting prior topic knowledge and strength of internal tag representations

To elicit students’ prior knowledge about particular concepts of the topics to be investigated in SOBOLEO (control variable) and students’ internal representations of shared tags (dependent variable), we applied a free association test utilizing (a sample of) tags as open probes. The students were asked to write down all associations for each of the sampled tags coming to their mind for 60 s. After excluding repetitions, we used the number of associations as an indicator for the strength of representation of a tag in students’ semantic memories (e.g., Srinivas & Roediger, 1990; Weldon & Coyote, 2006). The free association test is a standard task assessing association behavior (e.g., Benedek, Könen, & Neubauer, 2013). It elicits situational knowledge about concepts and provides a realistic account of human knowledge about the concept (Morais, Olsson, & Schooler, 2010)

We applied the free association test at the beginning, at half time and at the end of the study. At the beginning, we used it to elicit students’ prior knowledge about the topics and to examine whether different experimental groups were equivalent with respect to prior knowledge (see below). Since at that time no tags had been generated, we chose five concepts (“social software”, “wiki”, “knowledge wiki”, “weblog” and “project weblog”) as open probes that we deemed central for the two topics the students were later asked to work on. At half time and at the end, we randomly drew tags from different levels of the taxonomies the students had generated up to the given point in time and used these tags as open probes.

#### 3.2.3 FIDEC III: Attitudes towards computer as a means for collaboration

As a further control variable we used the six items sub-scale FIDEC III of the standardized inventory INCOBI (Nauman, Richter, & Groeben, 1999) to elicit attitudes towards the computer as a communication instrument (e.g., *For me it’s important to exchange views with friends per computer*) using a 5-point-Likert scale from *agree* to *do not agree*. This scale shows good internal consistency (Cronbach’s alpha = .86).

#### 3.2.4 Self-reported topic understanding and satisfaction with collaboration

At the end of the study, the students were given a self-developed, five items questionnaire on their general understanding of the topic (Item1: *The task helped me to gain a proper understanding of the topic ‘Wikis in enterprises’/‘Weblogs in university courses’*), satisfaction with the SOBOLEO system (Item 3: *I was well supported by the software SOBOLEO in fulfilling my desired working steps*, and Item 2: *The relations ‘broader’, ‘narrower’ and ‘related’ of the taxonomy editor were sufficient to create a meaningful taxonomy*), the communication within the group (Item 4: *I’m satisfied with the communication within my group in the SOBOLEO environment*) as well as the shared taxonomy (Item 5: *I’m satisfied with the collaboratively created taxonomy in SOBOLEO*), using a 5-point-Likert-scale from *agree* to *do not agree* as well as an open-ended question (*Provide some reasoning for your answer*). An item analysis revealed a relatively high item difficulty indicating a general tendency of the students to answer the corresponding statements in the negative direction (*p*_item1_ = 0.3, *p*_item2_ = 0.18, *p*_item3_ = 0.5, *p*_item4_ = 0.10, *p*_item5_ = 0.36). A test of normal distribution by means of the *z-*standardized measures of skewness and kurtosis revealed that – except for item 3 – the critical value of *z_crit_ *= 2.58 was not exceeded (item 1: *S *= −0.92, *K *= −0.66; item 2: *S *= 0.11, *K *= 0.95; item 3: *S *= −3.61, *K *= 5.71; item 4: *S *= 2.11, *K *= −0.20; item 5: *S *= −0.66, *K *= −0.13). Taken together, we regarded items 1, 2, 4 and 5 as appropriate to differentiate between students’ opinions on the task and software and to better understand results of our statistical analyses.

### 3.3 Independent variables

#### 3.3.1 Topic duration

**Fig. 3:**
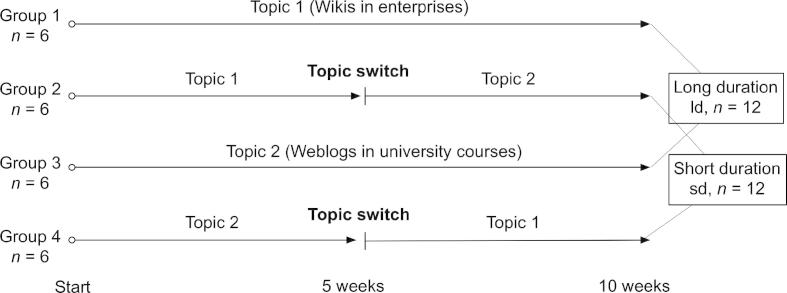
Variation of topic duration.

Fig. 3 shows the procedure applied to vary topic duration to observe its impact on semantic stabilization and tag specificity. First, from the sample of *N *= 24 students, we formed four *n *= 6 student groups, each working in a separate SOBOLEO instance, in the following way. After conducting the free association test to elicit students’ prior knowledge (see above), we ranked them according to their average number of unique associations to the five open probes. Then, the first four ranked participants were each randomly assigned to one of the four groups. This procedure was repeated for all remaining quadruples such that the final four groups were equivalent according to the free association scores (*F*_3,20_ = 0.61, *n.s*.). Additionally, we checked for equivalence with respect to the FIDEC III scale (*F*_3,20_ = 0.14, *n.s.*).

We assigned two groups (1 and 3) to the long duration (*ld*) and the other two groups (2 and 4) to the short duration (*sd*) condition. Under the *ld condition*, each of the two groups of students worked collaboratively on one topic (either on “Wikis in enterprises” or on “Weblogs in universities”) for the whole study duration (10 weeks). Under the *sd condition*, they had to switch the topic (either from topic 1 to 2 or from topic 2 to 1) at half time and, thus, worked on each topic for only five weeks. To control for the development of skills in using the tagging technology and for the formation of social roles, we restricted data analyses to the second half of the study, i.e., to weeks 6–10. We pooled all data of the *ld condition* (i.e., data generated by the groups 1 and 3) and the *sd condition* (i.e., groups 2 and 4). That way, we could observe the effect of topic duration independent of topic and topic sequence.

As described above, we also conducted the free association test at half time to test the assumption that up to this point in time no group differences should exist due to similar experimental conditions. We utilized a sample of randomly drawn tags as open probes where participants were only given tags from their own SOBOLEO instance. The means and standard deviations for the *sd* and *ld condition* were *M*_sd_ = 3.61, SD_sd_ = 0.52 and *M*_ld_ = 3.57, SD_ld_ = 1.12, respectively. Since the assumption of equal variances was not met (according to the Levene test) we performed a Welch’s *t*-test. In accordance with our expectation, there were no significant differences in the average number of associations between the two conditions, *t*_15.3_ = .09, *n.s*. We are therefore confident that potential differences in semantic stabilization and tag specificity that might turn out in the course of the second study period (weeks 6–10) can be attributed to the topic switch at half time, i.e., to the independent variable of topic duration.

#### 3.3.2 Tag specificity

We utilized the tag taxonomy of each SOBOLEO instance to vary *tag specificity*, the second independent variable. It was not possible to use a categorization norm to distinguish between superordinate, basic and subordinate categories because the tags generated by the students (e.g., *videoblogs*) were not available in such norms. Therefore, we decided to differentiate tag specificity by considering tags of the first SOBOLEO taxonomy level as general (e.g., *weblogs, e-learning by collaborating*), tags of the second level as medium (e.g. *types of weblogs, psychology of weblogs*) and tags of levels below the second one as specific (e.g. *videoblogs, microblogging*). Table 1 shows the number of tags drawn from each level in the corresponding SOBOLEO instance (group). Numbers in brackets represent the total number of available tags per level. From each of the four taxonomies, we only drew three general tags since – except for the group 2 taxonomy – only three were available. Independent of the taxonomy, we randomly sampled eight medium and eight specific tags.

To validate the assumption that the general, medium and specific tags vary in specificity, we firstly checked the number of bookmarks connected to these tags in SOBOLEO. In fact, general tags were associated with a higher number of resources (*M* = 9.82, SD = 2.99) than medium tags (*M* = 4.92, SD = 2.30) and specific tags (*M* = 2.54, SD = 1.69) and hence, exhibited a lower information value (Halpin, Robu, & Shepherd, 2007). As a second validation check, we conducted a Google search using the tags as search terms. Indeed, we found that the number of search results decreases with increasing tag specificity (*M*_general_ = 7,825,918; SD_general_ = 12,295,281; *M*_medium_ = 2,729,520; SD_medium_ = 9,643,332; *M*_specific_ = 788,157; SD_specific_ = 2,683,861) again suggesting that general tags exhibit lower information values than medium and specific tags. We take these results as substantiating our use of the SOBOLEO taxonomy as an operationalization of tag specificity.

### 3.4 Dependent variables

#### 3.4.1 Semantic stabilization: Number of unique tags as a function of time and topic duration

From week 6 to 10, we calculated the number of unique tags *N_u_* of the tag distribution at each time step *t* of a tag assignment, i.e. where a student had either reused an existing tag or added a new tag to her/his SOBOLEO instance. For the sake of comparability, we restricted the analysis to the sequence of consecutive tag assignments from time step *t*_1_ to *t*_165_, since in each group at least 165 tag assignments had taken place in the second half of the study.

#### 3.4.2 Tag use as a function of time and specificity

To measure tag use, we considered all tag assignments (using new tags or reusing existing ones) in the second 5-week period under the *ld* and *sd condition* and noted for each tag assignment, whether the used tag was general, medium or specific. To obtain usage frequencies as a function of time, specificity and topic duration, we then counted the number of used tags for the three specificity levels separately for each of the five weeks (weeks 6 to 10) under the *ld* and *sd condition*. That way we obtained a three-way 3 (levels) × 5 (weeks) × 2 (topic durations) contingency table.

#### 3.4.3 Strength of internal representations of tags

We applied the free association test utilizing tags as open probes (see materials) and the number of unique associations as an indicator for the strength of internal tag representations.

### 3.5 Design

The independent variables formed a mixed 2 × 3-design constituted by the factors *topic duration* (long duration vs. short duration, between-subjects) and *specificity of tags* (general vs. medium vs. specific, within-subjects). The main dependent variables were (i) the parameters of equation 2 as a measure of semantic stabilization (i.e., the asymptote *H* and the rate of approaching the asymptote *b*), (ii) the frequencies of tag use and (iii) the strength of internal representations of tags.

To test hypothesis H1 (that the semantic stabilization of the tag distribution is more pronounced under the *ld* than under the *sd condition*), we merged the *N_u_(t)* distributions of the groups 1 and 3 (*ld condition*) and of the groups 2 and 4 (*sd condition*) to get an average distribution as a function of topic duration, *N_u_*(*t*)*_ld_* vs. *N_u_*(*t*)*_sd_*. Then, we determined the approximately best fitting cumulative exponential given by Eq. (1) (Section 2.1) for *N_u_*(*t*)*_ld_* and *N_u_*(*t*)*_sd_* separately using Maximum Likelihood Estimation. This procedure resulted in estimates of the asymptote *H* (amount of agreement in using tags given unlimited time) and the slope *b* (rate of approaching the asymptote) for the *ld* and *sd condition*. Besides contrasting *H* and *b* of the two conditions, we tested the differences between *N_u_*(*t*)*_ld_* and *N_u_*(*t*)*_sd_* performing a Mann–Whitney-*U*-Test. We applied this non-parametric test since we expected dramatic deviations from a normal distribution (see Eq. (1)). Given statistical error probabilities *α *= *β* = .05 and the sample size *N *= 165 (time steps; see 3.4.1), we could detect an effect size of *f *= 0.37.

To test hypothesis H2.1 (that groups exhibiting stronger semantic stabilization apply tags from the medium and specific taxonomy levels more frequently than groups exhibiting weaker stabilization), we further processed the three-way 3 (levels) × 5 (weeks) × 2 (topic durations) contingency table. In the following we denote these three factors *L*, *W* and *T*, respectively. First, to examine the general assumption that *L* and *W* are associated under both topic duration conditions (i.e., that under both conditions, tags become more specific in time), we performed a *χ*^2^ tests on the marginal *L *× *W* table. Given statistical error probabilities *α *= *β* = .05, *df *= 8, and the total sample size *N *= 548, we could detect an effect size of *w *= 0.20. Second, we examined as to whether the association (joint distribution) of *L* and *W* changes as the level of *T* changes. We therefore tested the fit of a model assuming independence of the joint distribution from *T*. In case of a significant deviation of the joint independence model from the empirical data (i.e., the saturated multinomial model), it can be concluded that the association differs between the two groups. The fit of the model was evaluated performing a log-linear model with the joint independence assumption (*LW*/*T*).

With respect to the third dependent variable (strength of internal tag representations), per participant we calculated a mean free association score for each of the three taxonomy levels. For instance, if a participant produced 4, 6 and 4 unique associations to the three tags drawn from the first, i.e., general level of the corresponding SOBOLEO taxonomy (see Table 1), the participant’s mean free association score for the general level would be 4.67. To test hypothesis H2.2 (that groups exhibiting stronger semantic stabilization produce more associations to medium and specific tags than groups exhibiting weaker stabilization) we performed a 2 (topic duration) × 3 (tag specificity) ANOVA for repeated measures on the free association scores. Given statistical error probabilities *α *= *β* = .05 and *N *= 24, we could detect an effect size of *f *= 0.34.

### 3.6 Procedure

In the first course unit, an introduction to SOBOLEO was provided and students completed the free association test (see Section 3.2.2) and filled in the INCOBI subscale FIDEC III (see Section 3.2.3). These scores were used to ensure that the four *n *= 6 student groups were equivalent with regard to their attitudes towards the computer as a communication means and prior knowledge (see Section 3.3.1).

After the first course unit, each group was provided with their own SOBOLEO instantiation only accessible by personal usernames and passwords. E-mails were sent out to inform the participants of the topic they had to work on with access details for their SOBOLEO environment. Two groups were asked to research the topic “Wikis in enterprises”, the other two groups “Weblogs in university courses”. They were asked to prepare these topics as if they were collaboratively working on a report of presentation. The participants did not know who their fellow group members were and they worked on these assignments from home without meeting their fellow group members in person. Both topics were chosen because they were related to the course subject and because we expected the participants to have only little prior knowledge about them.

During the whole duration of the study (ten weeks) each student was expected to post two relevant bookmarks per week to the SOBOLEO environment and describe them with meaningful tags. The students were also required to collaboratively organize their tag collection with the help of the SOBOLEO taxonomy editor. To facilitate the emergence of consensus, the students were also encouraged to utilize the SOBOLEO chat and an external discussion forum.

After five weeks (at halftime), the SOBOLEO environments of two of the four groups were cleared. They had to start from scratch and to work on the other topic for another five weeks, making them the groups of the *short duration (sd) condition*. The other two groups continued with their prior topic in the *long duration (ld) condition*. The second five week period was thus the crucial experimental period in which all measures were taken. As reported in Section 3.3.1, students under the *sd* and *ld conditions* were still equivalent with regards to their knowledge of the topics at the beginning of this period.

At the end of the study after the second five week period, the free association test was administered. Also participants were presented the 5-items questionnaire on their general understanding of the topic and their satisfaction with the collaboration within the group.

## 4 Results

### 4.1 The influence of topic duration on semantic stabilization (Hypothesis H1)

**Fig. 4:**
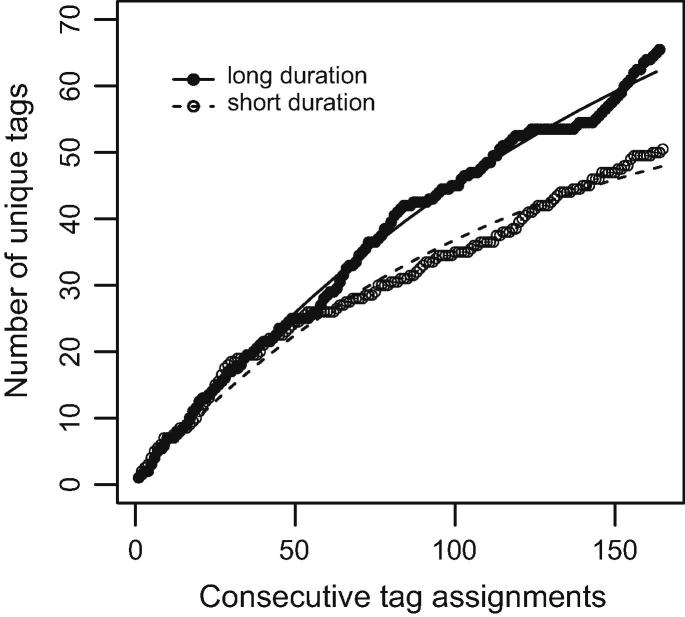
Semantic stabilization as measured by number of unique tags in the *ld* and *sd condition* in the second half of the study.

Regarding hypothesis H1, Fig. 4 shows the *N_u_(t)* distributions (number of unique tags as a function of consecutive tag assignments) for the *sd* condition (unfilled circles) and the *ld* condition (filled circles) as well as the respective, approximately best fitting exponential function given by Eq. (1). The amount of variance explained by Eq. (1) is *R*^2^ = 0.97 (for the *sd condition*) and *R*^2^ = 0.95 (for the *ld condition*). A glance at the figure reveals that topic duration (independent variable) had a strong impact on semantic stabilization since descriptively, the asymptote *H* and rate of approaching the asymptote *b* differ between the two conditions. However, in contrast to our expectation, semantic stabilization (in terms of a small estimate of *H* and a relatively larger estimate of *b*) appears to be more pronounced under the *sd* than under the *ld condition* (*H_sd_ *= 61.81, *b_sd_ *= 0.009; *H_ld_ *= 98.86, *b_ld_ *= 0.006). In particular, from the 60th tag assignment, the number of unique tags is lower under the *sd* than under the *ld condition*. In accordance with this descriptive data analysis, the Mann–Whitney-*U*-test yielded a highly significant difference between the two *N_u_(t)* distributions (*W *= 10293.5, *p *< 0.001).

This result suggests that there were different levels of semantic stabilization under the two conditions (short and long duration). However, despite the fact that groups in the *ld condition* had already worked on the same topic in the previous five weeks, they exhibited a slower rate of stabilization and were less in agreement on the use of tags in the second 5-week period than the *sd* groups.

To explain this unexpected result, we looked at results of the post hoc questionnaire that had been administered to the students at the end of the semester. First, all groups indicated they had been dissatisfied with the communication mechanisms (the SOBOLEO Chat and discussion forum). Albeit having worked on their topic for a longer time, students in the *ld condition* gave significantly lower ratings when asked for the understanding of the topic (*M* = 1.67 on a 5-point Likert scale, SD = 1.23) than students in the *sd condition* (*M* = 2.69, SD = 0.75; *F*_1,23_ = 6.44, *p* < .05). Additionally, *ld groups* (*M* = 1.92, SD = 1.00) perceived a lower quality of their taxonomy than *sd groups* (*M* = 2.92, SD = 0.86; *F*_1,23_ = 7.33, *p* < .05). Free text answers indicated that especially students in *ld groups* found it more difficult to collaboratively work on the shared taxonomy in SOBOLEO and they felt that their collaboration had resulted in a chaotic collection of bookmarks and tags where it was rather difficult to keep an overview. Moreover, the discussion forum that had been provided for discussing the shared taxonomy was only used occasionally.

In conclusion, while our experimental manipulation (duration of engagement with a topic) was obviously effective in producing differences in semantic stabilization in the two conditions as measured by convergence time, it was actually the students working under the *sd condition* that converged more quickly and more successfully than those in the *ld condition*. We suspect that the reason for this was that because all students were very inexperienced in collaborative tagging, the first five weeks served as a kind of trial period in which students had to learn about how to tag resources effectively and how to build up an effective taxonomy. And while after those five weeks the *sd groups* were able to start anew because their environments (and the built taxonomy) were cleared at half-time, the *ld groups* had to continue using the taxonomy they found unhelpful. This obviously helped the students in the *sd groups* to build a more effective and shared tag taxonomy in the second half of the study and converge to a common tag vocabulary more quickly. The negative effect for *ld groups* was exacerbated by missing effective direct communication mechanisms in the SOBOLEO system. Further results that we do not report here in more detail also support this interpretation, e.g. that the number of tags that were shared within a group (i.e. used by more than just one person) were much higher in the *sd* groups than in the *ld* groups.

Because we were successful in manipulating the process of semantic stabilization, i.e., *H* and *b*, we continue our statistical analyses testing H2.1 and H2.2 in the following sections. We assume that the faster rate of semantic stabilization (under the *sd condition*) led to a higher usage frequency and a stronger internal representation of medium and specific tags.

### 4.2 The impact of semantic stabilization on the specificity of tag assignments (Hypothesis H2.1)

According to hypothesis H2.1, we expected students of the *sd condition*, who had turned out to create a more stable tag vocabulary, to exhibit a stronger basic-level shift and thus, to apply medium and specific tags more frequently than students of the *ld condition*. The frequency analyses reported next are based on the three way 3 (levels) × 5 (weeks) × 2 (topic durations) contingency table (see Table 3 in the Appendix A). In a first step, we tested the general assumption of a basic-level shift (e.g., Tanaka & Taylor, 1991) independent of experimental condition, i.e., a constant use of general tags accompanied by an increasing dominance of medium and specific tags to categorize and label bookmarks in SOBOLEO.

To this end, we gathered the marginal frequency table of *tag specificity* (general, medium, specific) and time (weeks 6–10) summing over topic duration (*sd* and *ld condition*). The relative row frequencies are presented in Table 2. While general tags have been applied at a fairly stable rate of around 0.20 across the five weeks, there appears to be an upward trend in the use of medium and specific tags (except for week 8). A 3 × 5 *χ*^2^- test on this marginal table yielded a significant deviation from data that would be expected under the null hypothesis of no association between week and specificity (*χ*^2^(8) = 18.15, *p* < .05). Hence, we conclude that students applied general tags constantly and tended to increasingly apply medium and specific tags across the five weeks.

**Fig. 5:**
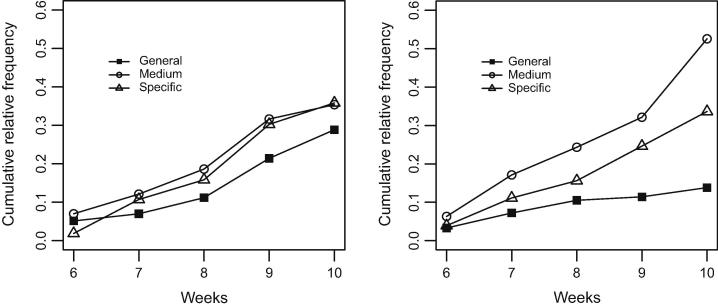
Specificity of tag assignments over time under the *ld condition* (left panel) and *sd condition* (right panel).

In a next step, we examined the whole three way contingency table to test H2.1, i.e., as to whether the basic-level shift revealed by Table 2 varied between the two conditions of differing semantic stabilization. Fig. 5 contrasts the group-specific tag use frequencies for each of the five weeks and the three specificity levels. To better depict the temporal dynamics in using tags on the three levels, the figure shows cumulative frequencies.

The pattern shown in Fig. 5 clearly speaks in favor of our assumption that groups developing a more stable tag vocabulary for sharing bookmarks (i.e., groups under the *sd condition*) exhibited a stronger basic-level shift. While under both conditions, medium and specific tags soon started dominating general tags for the categorization of bookmarks, the continuously increasing importance of medium and specific tags was much more pronounced under the *sd condition*. The log-linear model for the null hypothesis that the time-specificity association does not differ between the two conditions provided a poor representation of the observed data (*Pearson*’s *χ*^2^ (14) = 40.46, *p* < .001). Therefore, our results do not allow for rejecting H2.1 and support the assumption that conditions conducive for semantic stabilization (in the *sd condition* in our case) amplify the basic-level shift for all group members.

### 4.3 The impact of semantic stabilization on memory representations of tags (Hypothesis H2.2)

H2.2 postulates that the basic-level shift, which we assume to depend on the semantic stabilization, does not only become manifest in a particular pattern of tag use but also in relatively strong memory representations (number of unique associations) for medium and specific tags. In particular, H2.2 assumes an interaction between topic duration and tag specificity with respect to memory strength: While students under the *sd* and *ld condition* should on average generate an equal number of free associations to general tags, *sd* students should generate more associations to medium and specific tags than *ld* students.

**Fig. 6:**
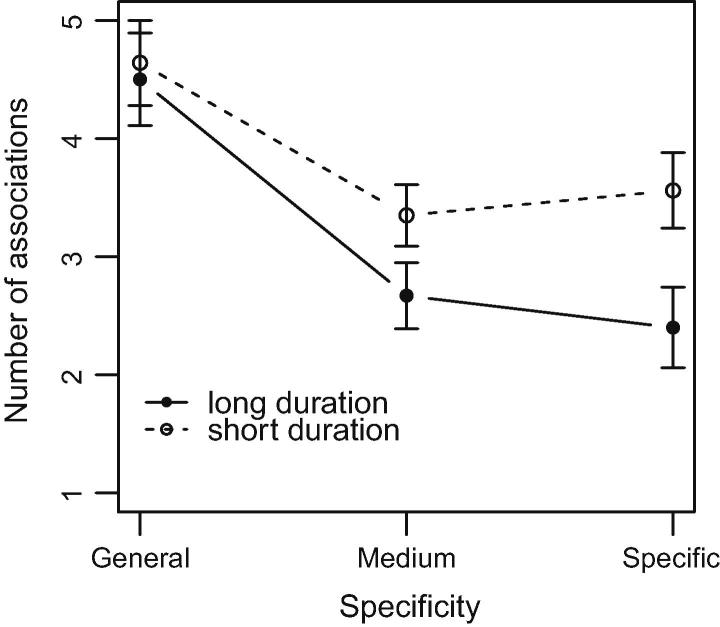
Number of unique associations in the *sd* and *ld condition* to general, medium and specific tags in the free association test.

Fig. 6 shows the mean free association scores (and standard deviations) for the two groups on the three tag specificity levels. By depicting an interaction between specificity and topic duration it reveals a pattern in line with our expectation: The large overlap of error bars on the general level of specificity indicates only small differences in the mean association scores between the two conditions (*M*_sd_ = 4.64, *SD*_sd_ = 0.36; *M*_ld_ = 4.50, *SD*_ld_ = 0.39). The non-overlapping error bars for the lower two levels of specificity, on the other hand, suggest that students under the *sd condition* generated more associations to medium and specific tags (medium: *M *= 3.35, *SD *= 0.26; specific: *M *= 3.56, *SD *= 0.32) than students under the *ld condition* (medium: *M *= 2.67, *SD *= 0.28; specific: *M *= 2.40, *SD *= 0.34). The 2 × 3 ANOVA with repeated measures supported this descriptive analysis yielding a main effect for specificity (*F_2_*_,21_ = 38.01, *p* < .001) and a significant interaction between specificity and topic duration (*F*_2,21_ = 5.06, *p* < .05). A main effect for topic duration could not be identified (*F*_1,22_ = 3.46, *n.s.*).

## 5 Discussion

The results we have reported have a number of implications for research in web science, in cognitive science and for education. Here we discuss three of them. Firstly, the results uncover the exact mechanisms of semantic stabilization that have so far been studied in web science research by statistical modeling of large-scale web environments without direct access to the users’ cognitive mechanisms. Secondly, the result point to the large influence that social mechanisms on the group level (here semantic stabilization mediated by shared artefacts) has on individual categorization which can be potentially overlooked if cognitive science research is conducted in the lab, rather than in real-life settings. And thirdly, we discuss several educational implications of collaborative tagging that our study suggests. We now discuss each of these in turn.

### 5.1 Implications for web science research: Semantic stabilization and individual learning contribute to improved information value of social tags

Our study is in the tradition of research attempting to uncover the dynamics in collaborative tagging systems. Here we have focused on semiotic dynamics in the form of semantic stabilization, i.e. an emerging consensus on particular terms over time that can be observed despite the missing central control. Semantic stabilization has been previously studied in large scale tagging systems by modeling the emergent consensus by means of statistical models (Cattuto, Loreto, & Pietronero, 2007; Dellschaft & Staab, 2008; Halpin et al., 2007; Wagner et al., 2014). In this paper, we complement this research by studying some of the micro-level mechanisms (e.g. cognitive level phenomena of categorization). At the same time, experimental studies using collaborative tagging environments, such as the one conducted by Held et al. (2012), have studied individual level behaviors (navigation and incidental learning) and how these are influenced by collective knowledge (presumably the tag clouds captured in those environments). Because these studies limit the social interaction between users of the system, it is not possible to study the emerging consensus in a community of users that would lead to semantic stabilization.

By conducting a field experiment that has allowed us to track categorization over time, not only by looking at the use of tags, but also examining the representation of those categories in memory, our study fills the gap between these two perspectives. This allows us to explain how semantic stabilization and individual learning processes play together to produce emergent characteristics of collaborative tagging systems. Specifically, we assume that a driving force behind the processes of semantic stabilization and the basic-level shift is that our students were trying to optimize the information value of the tags they used both for browsing and for indexing resources. Halpin et al. (2007) define the information value of a tag by the number of resources it returns, where the smaller the number, the higher the information value. The level of abstraction of a category is related to the information value as a tag that corresponds to a general category would return a high number of resources, while more specific tags would return lower number of resources, making their information value higher. We show empirically that tags from different levels of the SOBOLEO hierarchy correspond to different levels of information value. More specific tags returned fewer resources collected by the students in the study, and they also returned fewer resources when employed as search terms in a search engine (see Section 3.3.2).

In a sensemaking task employing social tags, a successful system should tend towards tags with a higher information value. In our study, we show that an important prerequisite for reaching a higher information value of more specific categories and their corresponding tags is that semantic stabilization takes place. Semantic stabilization facilitates a quicker shift to more specific levels of abstraction and therefore to a higher information value. In the *sd* groups, negotiations on the group level have led to a more optimal information value than in the *ld* groups and this was also related to higher levels of individual learning (as self-assessed by the students and confirmed with higher numbers of unique associations in the association test) and to higher degrees of satisfaction with the constructed taxonomy.

We intend to employ some of the insights we gained from this study to develop recommendation mechanisms that support more effective collective learning in the context of collaborative tagging. For example, Seitlinger, Kowald, Trattner, and Ley (2013) suggest a tag recommender that is based on models of human categorization which proved to predict application of existing tags consistently and more effectively than other approaches. The next goal would now be to apply insights from the present study to improve semantic stabilization with recommender mechanisms, e.g. including a mechanism that is more sensitive to base level categories and their shift over time.

### 5.2 Implications for cognitive science research: Individual categorization is mediated by collective phenomena and shared artefacts

From a cognitive science perspective, our study shows that formation and variation of individual categorization in sensemaking is mediated by collective phenomena on a group level. In contrast to traditional categorization research which is predominantly conducted in laboratory settings, the present study therefore adds an innovative aspect as we show that individual expertise is to some extent dependent on the semiotic dynamics on a group level. By varying the amount of opportunity in which groups can negotiate a common meaning around the use of artefacts (“Convergence Time”, Steels, 2006), we have realized a situation in which natural emergence of categories can be observed in the wild, rather than in a laboratory induced way (Glushko, Maglio, Matlock, & Barsalou, 2008). In our study, semantic stabilization as a phenomenon on the group level mediated categorization on an individual level. Students in the short duration group were faster to establish a shared vocabulary, and this contributed to the formation of more refined categories on more specific levels of abstraction (basic-level shift) indicating that they established higher levels of expertise more quickly than the students in the long duration groups.

The study also speaks for the important role of shared artefacts in collaborative human activity. Distributed cognition theory (e.g. Hollan, Hutchins, & Kirsh, 2000) assumes, external representations and internal representations are coordinated and mutually influence each other (Hutchins & Hazlehurst, 1995). Our results show that both internal representation of tags (the memory measures) as well as the external representation (the tags students used) were in agreement to show that *sd* groups shifted to more specific levels of abstraction, both in the tags they applied as well as in the internal cognitive categories they used.

The important role of artifacts in human activity and sensemaking is also proposed by a postphenomenological perspective on technology (Verbeek, 2005). Our study provides evidence for the active role of artifacts (e.g., tags and tag taxonomies) in shaping “the relation between humans and their world by mediating praxis” (Verbeek, 2005, p. 90), e.g., collecting and categorizing bookmarks. According to this view, networks of meaning crystallize around a concrete thing in a cognitive ecology as a result of dynamic feedback processes. Stronger internal associations are reflected in the shared artefact which in turn strengthens the internal representation. We demonstrate here that these processes can be observed in the natural context of a university course and our results emphasize that an important mechanism leading to the establishment of networks of meanings in artefact-mediated collaboration is semiotic dynamics that leads to a semantic stabilization over time.

The fact that short duration groups were quicker to find consensus is a counterintuitive result. Obviously, the tag collection and the shared taxonomy that the students built seemed to play a more important role for learning than the amount of time students were engaged in the topic. The collaboratively built taxonomies had a significant and differential influence on the development of categories of the *sd* and *ld groups*: Despite the fact that *ld groups* engaged twice as long with the topic than *sd groups,* it was the *sd groups* that showed higher degrees of subjective learning and were more confident with the results (the taxonomy that was generated) than *ld groups*. Additional results we have not reported here point to the fact that the reuse rate of tags (reapplying existing tags from one’s own or others’ vocabulary) was much higher in *sd groups* than *ld groups*. This gives further evidence that the external representation was more useful for *sd groups* as compared to *ld groups* and provided a better means for learning.

Our study also shows the importance of moving cognitive science research out of the laboratory into the “real world” because it points us to phenomena that may be overlooked in the lab (Glushko et al., 2008; Hintzman, 2011). For example, communication plays an important role in developing categories (Gelman, Coley, Rosengren, Hartman, & Pappas, 1998) and communication is often mediated through the use of artifacts (Hutchins & Hazlehurst, 1995; Stahl, 2002). Although the important role that external artefacts play for cognitive processing has often been stressed in cognitive science (e.g. Zhang, 1994), it is generally overlooked in categorization research when it is purely lab based. Our study shows the tremendous opportunity that exists for the study of human cognitive processing when it employs social web tools. After all, our results were partly unexpected because of the particular dynamics of the collaborative situation.

### 5.3 Implications for education: Collaborative knowledge building and the coordination of internal and external representations

The effectiveness of the shared tag collection together with the taxonomy obviously had a stronger effect on learning than the duration students engaged with the particular topic. Quite clearly, the quality of the learning interaction was more important than the quantity. Recently, Cress et al. (2013) have suggested that individual knowledge and collective knowledge (represented by the tags used) are co-evolving in a collaborative learning task when shared artefacts are used. The co-evolution model attributes the emergence of knowledge structures in the Web to a continuous interaction between internal (mental) processes and socially constructed external artifacts, such as tags and collaboratively constructed taxonomies as in our case. With our study, we contribute to this perspective by studying the social processes that are assumed to underlie the formation of collective knowledge. In our study, we allowed the emergence of collective knowledge (semantic stabilization) to happen as part of the study design and tracked it over time.

We also showed that a stable interpretation needs to develop in a community of learners as a result of students using shared artefacts before individuals can benefit from the collective knowledge. A surprising result was also that the shared artefact in our study was actually hindering learning in the *ld* groups. Obviously it is sometimes better to get rid of a shared representation altogether if it proves unhelpful for learning (as we did in the *sd* groups). From the perspective of the co-evolution model, it could be argued that accommodation of knowledge was not possible in the *ld* groups as the collective knowledge was not able to support this kind of operation.

From an educational point of view, recent research has shown that collaborative tagging can support students in learning categories in an exploratory learning task in the classroom and beyond (Kuhn, Cahill, Quintana, & Schmoll, 2011; Kuhn et al., 2012). Our study shows quite clearly that effective communication mechanisms need to be in place to enable collaborative learning. In the version of SOBOLEO we had used in the study, students’ chat messages were not persistent which drastically reduced usefulness of the chat for discussing about the use of tags. As this had been anticipated, we had set up an additional discussion forum for students to use. However, this forum was not used very much because it was outside the SOBOLEO environment. In current versions of the SOBOLEO tool, these shortcomings have been corrected. In similar studies of tagging systems in educational settings (Kuhn et al., 2012; Yew et al., 2006), authors usually combine tagging activities with face to face interaction which greatly enhances the benefit for learning purposes.

## 6 Limitations and future work

Semantic stabilization is a group process that we assume is dependent on a number of factors. Here we only studied two of them, namely the time of engagement with a topic and the effectiveness of the shared representation. We found that the latter was a much stronger factor in producing convergence than mere collaborative study time. Future studies need verify this result and also disentangle other factors that influence semantic stabilization, such as the trustworthiness of the other persons, the quality of interaction, as well as the design of the tagging environment (such as the type of tag recommendations or how shared tags are displayed in the environment).

## Author disclosure statement

No competing financial interests exist.

## Figures and Tables

**Table 3:** Tag use frequencies depending on topic duration, specificity and time.

Topic duration	Tag specificity	Week					Total
6	7	8	9	10
sd	General	11	13	11	3	8	46
Medium	21	36	24	26	68	175
Specific	13	24	15	30	30	112
Total	45	73	50	59	106	333

ld	General	11	4	9	22	16	62
Medium	15	11	14	28	8	76
Specific	4	19	11	31	12	77
Total	30	34	34	81	36	215

**Table 1:** Variation of tag specificity: Tag samples drawn from the three levels from each of the four SOBOLEO taxonomies.

		SOBOLEO instance (group)				∑
		1	2	3	4	
Level	General	3 (3)	3 (5)	3 (3)	3 (3)	12 (14)
Medium	8 (15)	8 (23)	8 (21)	8 (30)	32 (89)
Specific	8 (50)	8 (16)	8 (14)	8 (30)	32 (110)
Sum	19 (68)	19 (44)	19 (38)	19 (63)	76 (213)

*Note.* Numbers in brackets represent the total number of available tags on each level.

**Table 2:** Frequency of tag use on three levels of specificity in the second half of the study period (weeks 6–10).

Specificity	Week					Total
6	7	8	9	10
General	0.20	0.16	0.19	0.23	0.22	1
Medium	0.14	0.19	0.15	0.22	0.30	1
Specific	0.09	0.23	0.14	0.32	0.22	1

## Appendix A

See Table 3.
